# 1,25-(OH)_2_D_3_/Vitamin D receptor alleviates systemic lupus erythematosus by downregulating Skp2 and upregulating p27

**DOI:** 10.1186/s12964-019-0488-2

**Published:** 2019-12-10

**Authors:** Dan Liu, Yu-Xuan Fang, Xia Wu, Wei Tan, Wei Zhou, Yu Zhang, Yan-Qing Liu, Guo-Qing Li

**Affiliations:** 1 Department of Pathology, Clinical Medical College, Yangzhou University, Yangzhou, 225000 People’s Republic of China; 2 Department of Rheumatology and Immunology, Affiliated Hospital of Yangzhou University, Yangzhou University, No. 368, Hangjiang Road, Yangzhou, Jiangsu Province 225000 People’s Republic of China; 3 Clinical Medical College, Dalian Medical University, Dalian, 116044 People’s Republic of China; 4 Medical College of Yangzhou University, Yangzhou, 225000 People’s Republic of China

**Keywords:** Systemic lupus erythematosus, 1,25(oh)_2_D_3_, Vitamin D receptor, Skp2, p27, Renal injury, Inflammatory factors

## Abstract

**Background:**

Recent evidence has suggested that the 1,25(OH)_2_D_3_/Vitamin D receptor (VDR) acts to suppress the immune response associated with systemic lupus erythematosus (SLE), a serious multisystem autoimmune disease. Hence, the aim of the current study was to investigate the mechanism by which 1,25-(OH)_2_D_3_/VDR influences SLE through regulating the Skp2/p27 signaling pathway.

**Methods:**

Initially, the levels of 1,25(OH)_2_D_3_, VDR, Skp2, and p27 were measured in collected renal tissues and peripheral blood. Meanwhile, the levels of inflammatory factors, biochemical indicators (BUN, Cr, anti-nRNP IgG, anti-dsDNA IgG) and urinary protein levels were assayed in in VDRinsert and VDR-knockout mice in response to 1,25(OH)_2_D_3_ supplement. In addition, the distribution of splenic immune cells was observed in these mice.

**Results:**

Among the SLE patients, the levels of 1,25(OH)_2_D_3_, VDR and p27 were reduced, while the levels of Skp2 were elevated. In addition, the levels of anti-nRNP IgG and anti-dsDNA IgG were increased, suggesting induction of inflammatory responses. Notably, 1,25(OH)_2_D_3_/VDR mice had lower concentrations of BUN and Cr, urinary protein levels, precipitation intensity of the immune complex and complement, as well as the levels of anti-nRNP IgG and anti-dsDNA IgG in SLE mice. Additionally, 1,25(OH)_2_D_3_ or VDR reduced the degree of the inflammatory response while acting to regulate the distribution of splenic immune cells.

**Conclusion:**

This study indicated that 1,25-(OH)_2_D_3_/VDR facilitated the recovery of SLE by downregulating Skp2 and upregulating p27 expression, suggesting the potential of 1,25-(OH)_2_D_3_/VDR as a promising target for SLE treatment.

## Background

Systemic lupus erythematosus (SLE) is a chronic multifactorial autoimmune disease, which involves genetic and environmental factors [1]. SLE is characterized by the production of antinuclear autoantibodies that form immune complexes in different organs, including the skin, joints, kidneys and brain, consequently promoting a chronic inflammatory response [2, 3]. A prior study has highlighted estrogen as a contributing factor accounting for the ten times higher incidence of SLE in women, particularly during the reproductive years [4]. Otherwise, the etiological factors of the disease still remain unclear, while endocrine, genetic and environmental factors such as drugs, smoking, ultraviolet radiation, infectious agents, as well as various exposure to chemical agents have been suggested to be potential risk factors [5]. SLE patients often display a wide range of clinical symptoms, ranging from mild joint and skin problems to severe multisystem dysfunction manifesting in renal, neurologic as well as cardiovascular disorders [6]. Therefore, it is necessary to further develop a better understanding of SLE for its early detection and improved treatment.

Vitamin D transmits signals to target cells through the vitamin D receptor (VDR), which is expressed in all immune cells, has been validated to exert a variety of immunomodulatory effects on the prevention of autoimmune diseases [7, 8]. Furthermore, 1,25-(OH)_2_D_3_, the active form of vitamin D in vivo has been reported to suppress cell proliferation [9]. Studies have demonstrated that regulatory T cells (Tregs) play a critical role against the progression of SLE symptoms because of their immune-suppressive functions [10, 11]. However, Skp2 affects cell cycle control and cell death, and its overexpression could decrease the suppressive effect of Tregs in the progression of autoimmune disorders [12]. Deficiencies of p27, a cell cycle inhibitor, not only reduce the activity and quantity of Tregs, but also induce lupus-like abnormalities [13]. Both Skp2 and p27 play vital roles in mediating the migration, proliferation and invasion of tumor cells as well as balancing immune tolerance in the development of autoimmune diseases [12, 14], while 1,25-(OH)_2_D_3_ is reported to reduce Skp2 protein expression in metastatic prostate cancer cells [15]. Recent evidence has indicated that the 1,25(OH)_2_D_3_ analogs mediate the upregulation of p27 in head and neck squamous carcinoma cells by downregulating Skp2, which leads to p27 stabilization [16]. Mycophenolate mofetil, an inhibitor of guanosine nucleotide synthesis in lymphocytes is useful for the symptomatic treatment of SLE, but there is no effective cure [17], and other drugs, such as corticosteroids, azathioprine, and hydroxychloroquine failing to adequately decrease the risk of organ-specific disease [18]. Thus, the central objective of the current study was to investigate the potential role of 1,25-(OH)_2_D_3_/VDR in the progression of SLE by regulating the Skp2/p27 signaling pathways.

## Materials and methods

### Study population

One hundred and forty-nine patients with incipient SLE were enrolled from March 2015 to April 2016 from the Clinical Medical College, Yangzhou University and Yangzhou First People’s Hospital, including 144 females and 5 males with a mean age of 32.72 ± 8.56 years (ranging from 11 to 74 years). All patients conformed to the diagnostic criteria for SLE of the American College of Rheumatology’s rheumatoid arthritis electronic clinical quality measures [19] revised in 1997. The exclusion criteria were as follows: 1) patients taking potentially interfering medication such as sex hormones or calcitriol; 2) artificially reducing the interference of light (light-dependent vitamin D formation); and 3) abnormal thyroid function, diabetes, tumors and other autoimmune diseases. In addition, 150 healthy controls were selected. The health controls were selected from people undergoing physical examination at Clinical Medical College, Yangzhou University and Yangzhou First People’s Hospital, with all participants well informed regarding the objectives of the experiment. There were no significant differences in gender and age between the incipient SLE patients and healthy controls. Peripheral blood samples and renal tissues from all subjects were collected, with the collected blood samples analyzed using a Japan MEK-7222 automatic blood analyzer within 1 to 4 h. The demographics data of the included subjects is depicted in Table 1.

**Table 1:** Demographics information about the incipient SLE patients and healthy controls

Subjects	Gender	Number of cases	Average age
Incipient SLE patients	Male	5	32.72 ± 8.56
	Female	144	
Healthy controls	Male	9	33.09 ± 10.14
	Female	141	

Note: *SLE* systemic lupus erythematosus

### Separation of CD4^+^ T cells

The collected samples were subjected to density gradient centrifugation on a Ficoll-isopaque (Lymphoprep). The residue of the brown-yellow layer of leukocytes was removed from the samples, and the peripheral blood mononuclear cells (PBMCs) were separated. CD25^+^ cells with removal of CD4^+^ T cells were used throughout the study to avoid the potential inhibition of CD25^+^ proliferation by CD4^+^ cells. We used a CD4^+^ CD25^+^ regulatory T cell isolation kit (130–091-301, Miltenyi Biotech, Bergisch Gladbach, Germany) to isolate CD4^+^ CD25^−^ T cells from PBMCs by negative selection, based on the manufacturer’s instructions. The protein expression of Skp2 and p27 in isolated CD4^+^ CD25^−^ T cells was detected by western blot analysis.

### Animal grouping

A total of 60 specific-pathogen-free MRL-LPr/LPr spontaneous SLE mice and 40 C57BL/6.lpr mice (half male and half female, 7–8 weeks old, weighing 19–23 g, Model Animal Research Center of Nanjing University, Nanjing, Jiangsu, China) were housed at 22–25 °C. MRL-LPr/LPr VDRinsert mice and normal C57BL/6.lpr VDR-knockout mice were developed as describes in Additional file 1: Figure S1 and identified by Beijing Biocytogen Co., Ltd. (Beijing, China). VDRinsert mice refer to the transgenic mouse model introducing Rosa26 locus into VDR gene. The mice were divided into the control group (C57BL/6.lpr mice without 1,25(OH)_2_D_3_ supplement), VDR^−/−^ group (C57BL/6.lpr mice, VDR-knockout, without 1,25-(OH)_2_D_3_ supplement), SLE group (SLE mice without 1,25-(OH)_2_D_3_ supplement), SLE + VD_3_ group (SLE mice with 1,25-(OH)_2_D_3_ supplement) and SLE + VD_3_ + VDRinsert group (SLE mice with VDRinsert and 1,25(OH)_2_D_3_ supplement), with 20 mice in each group. Mice received supplement of 1,25(OH)_2_D_3_ (the active form of VD_3_, D1530-1MG, Sigma-Aldrich Chemical Company, St. Louis MO, USA) via a gastric tube (5 μg/kg per day). The remaining mice were placed on a normal dietary regimen. Ten mice from each group were randomly selected and promptly euthanized for tissue analysis (recorded as 0 W), while the remaining mice were maintained for a 24-week period of feeding (recorded as 24 W).

### Specimen collection

During the 8th, 16th and 24th weeks of treatment, the mice were weighed and anesthetized with 3% pentobarbital sodium (30 mL/kg, Sigma-Aldrich Chemical Company, St. Louis MO, USA) dissolved in normal saline (Wuhan Boster Biological Technology Co., Ltd., Wuhan, Hubei, China) via an intraperitoneal injection. Next, venous blood was collected in heparinized tubes for biochemical assays. The bilateral kidneys of each mouse were removed, with one portion weighed and used for assay of inflammatory factors by reverse transcription quantitative polymerase chain reaction (RT-qPCR), enzyme-linked immunosorbent assay (ELISA) and western blot analysis. The remaining part of the kidney was fixed by immersion with neutral formalin solution, dehydrated using gradient alcohol, cleared by xylene and embedded in paraffin (Thermo Fisher Scientific, San Jose, CA, USA). Next, 5-μm kidney tissue slices were prepared for histopathological observation.

### ELISA assay

After dissolving standards, 100 μL portions were added to draw a standard curve on the instructions of the ELISA kit (eBioscience, San Diego, CA, USA). The samples to be tested (100 μL) were added to the wells for a preincubation at 37 °C for 90 min. The samples were incubated successively with 100 μL biotinylated antibody for 60 min at 37 °C, after which 100 μL enzyme conjugates were added under subdued light for a further 30-min incubation at 37 °C, followed by the addition of 100 μL substrate for a further 15-min incubation at 37 °C in the dark, whereupon the termination buffer was added. The optical density (OD) value of each tube at 450 nm was determined using a multi-functional microplate reader (BioTek Synergy 2, BioTek Instruments, Winooski, VT, USA) within 3 min of termination. The standard curve was plotted in accordance with the OD values, and the contents of the analytes including 1,25-(OH)_2_D_3_), interleukin-4 (IL-4), IL-10, IL-17, Interferon-γ (INF-γ), anti-nRNP IgG, and anti-dsDNA IgG in the plasma samples of mice were analyzed. Data are mean values from three independent experiments.

### RT-qPCR

The RNA of renal tissues and CD4^+^ T cells was extracted using the TRIZOL method (Invitrogen, Inc., Carlsbad, CA, USA). The concentration and purity of the collected RNA were measured using a NanoDrop2000 (Thermo Fisher Scientific Inc., Waltham, MA, USA). According to the gene sequences published in the GenBank database, PCR primers were designed using Primer 5.0 primer-design software (Table 2) and synthesized by Shanghai GenePharma Co., Ltd. (Shanghai, China). RT-qPCR was conducted on the ABI PRISM 7500 real-time PCR System (ABI Company, Oyster Bay, NY, USA). The reliability of the PCR results was evaluated using a dissolution curve, with the CT value subsequently obtained (the inflection point of amplification curve). The fold changes between the experiment group and the control group were determined based on the relative quantification 2-^△△Ct^ method [20]. Data are mean values from three independent experiments.

**Table 2:** Primer sequences for RT-qPCR

Gene		Sequence
Human VDR	Forward Primer	5′-AGCTGGCCCTGGCACTGACTCTGCTCT-3′
	Reverse Primer	5′-ATGGAAACACCTTGCTTCTTCTCCCTC-3′
Human Skp2	Forward Primer	5′-GCTGCTAAAGGTCTCTGGTGT-3′
	Reverse Primer	5′-AGGCTTAGATTCTGCAACTTG-3′
Human p27	Forward Primer	5′-TAGTCAAAGTGCGAGTGTC-3′
	Reverse Primer	5′-TCTGTAGTAGAACTCGGGCAA-3′
Human β-actin	Forward Primer	5′-CATTGCCGACAGGATGCA-3′
	Reverse Primer	5′-CATCTGCTGGAAGGTGGACAG-3′
Mouse Skp2	Forward Primer	5′-ATGTGACTGGTCGGTTGC-3′
	Reverse Primer	5′-TCGATAGGTCCATGTGCT-3′
Mouse p27	Forward Primer	5′-GCCGAGATATGGAAGAAGCGA-3′
	Reverse Primer	5′-AAGAATCTCTGCCCGCAGGTCT-3′
Mouse β-actin	Forward Primer	5′-GCAGTTGGTTGGAGCAA-3′
	Reverse Primer	5′-ATGCCGTGGATACTTGGA-3′

Notes: *RT-qPCR* reverse transcription quantitative polymerase chain reaction, *VDR* vitamin D receptor, *Skp2* S-phase kinase-associated protein 2

### Western blot analysis

The proteins from the renal tissues of mice were extracted and analyzed with a bicinchoninic acid kit (Boster Biological Technology Co., Ltd., Wuhan, Hubei, China). The extracted protein samples were separated using 10% polyacrylamide gel electrophoresis (Boster Biological Technology Co., Ltd., Wuhan, Hubei, China), followed by transferring onto a polyvinylidene fluoride membrane. The membrane was blocked using 5% bovine serum albumin (BSA) for 1 h at room temperature and incubated overnight at 4 °C with the following primary antibodies to p-Skp2 (1: 1000, 14,865, Cell Signaling Technology, Beverly, MA, USA), Skp2 (1: 1000, ab183039, Abcam, Cambridge, UK), p-p27 (1: 1000, sc-12,939, Santa Cruz Biotech, Santa Cruz, CA, USA), p27 (1: 1000, ab193379, Abcam, Cambridge, UK) and β-actin (1: 1000, ab6276, Abcam, Cambridge, UK). Membranes were then washed and incubated with the secondary antibody for 1 h at room temperature and developed using chemiluminescent reagents. β-actin was regarded as the internal reference. The gray value of the target band was analyzed by Image J. Data are mean values from three independent experiments.

### Determination of biochemical indicators

The Coomassie brilliant blue method was applied to detect the urine protein content. The day prior to the end of the experiment (at the end of the 24th week), the mice were placed in metal metabolism cages for urine collection, followed by a centrifugation at 6000×g for 20 min and stored at − 80 °C. The urine protein was detected in accordance with the instructions of the kit (Nanjing Jiancheng Bioengineering Institute, Nanjing, Jiangsu, China). The BSA standard curve was subsequently plotted, followed by dilution of the supernatant with normal saline at a ratio of 1: 10. After the sample to be assessed was mixed with Coomassie Brilliant Blue dye at a ratio of 1: 10, the OD value at a wavelength of 540 nm was measured in an automatic microplate reader (Labsystems Dargon). After final urine collection, blood was collected retro-orbitally and subsequently stored for the assay of blood urea nitrogen (BUN) and serum creatinine (Cr) detection. The continuous monitoring method for urease was carried out in order to determine BUN. The reagents (10 μL) were mixed with 10 μL reagent provided in the kit (Rongsheng Biotech Company Co., Ltd., Shanghai, China) for the prompt colorimetric examination using a spectrophotometer (Thermo Fisher Scientific, San Jose, CA, USA). The absorbance values at 30 s (A1) and 90 s (A2) were then read to calculate BUN content using the formula: BUN (mmol/L) = (sample A2 - sample A1) / (blank A2 - blank A1) × (the concentration of the reagent). The content of serum Cr was detected using the picric acid method. Here, urine samples (100 μL) were mixed with an equal volume of the reagent provided in the kit (Rongsheng Biotech Company Co., Ltd., Shanghai, China) and water-bathed for 30 s at 37 °C. The absorbance values at 30 s (A1) and 90 s (A2) at 505 nm were then determined, and the concentration of Cr was calculated as follows: Cr (μmol/L) = (sample A2 - sample A1)/(blank A2 - blank A1) × (the concentration of the reagent). Data are mean values from three independent experiments.

### Hematoxylin and eosin (HE) staining and periodic acid Schiff (PAS) staining

The renal tissues were embedded in paraffin, dewaxed with xylene and hydrated with gradient alcohol (Boster Biological Technology Co., Ltd., Wuhan, Hubei, China). Then, the sections were stained with hematoxylin-eosin and PAS, and images of the section were captured under a microscope (CX31, Olympus Optical Co., Ltd., Tokyo, Japan).

### Immunofluorescence

Immunoglobulin G (IgG), immunoglobulin A (IgA), immunoglobulin M (IgM), complement 3 (C3) and complement (C1q) were labeled using fluorescein isothiocyanate (FITC). The renal tissue sections were dewaxed, treated with 3% catalase for 10 min for antigen retrieval, processed with pepsin for 10 min and sealed with fetal bovine serum (Gibco Company, Grand Island, NY, USA) for 1 h at 37 °C. Next, the sections were incubated with corresponding fluorescent antibodies against IgG (1:100), IgA (1:100), IgM (1:80), C3 (1:50) and C1q (1:50) (all of the antibodies and reagents were purchased from DAKO Company, Denmark) at 37 °C for 45 min. The sections were then washed and sealed with a buffered glycerol solution (DAKO Company, Denmark), whereupon the precipitation intensity of the immune complex was observed and photographed. The experiment was repeated three times.

### Detection of splenic immune cell proportions

Approximately 300 mg mouse spleen tissues were dispersed into a splenic cell suspension, followed by cell counting. The splenic cell suspension (1.0 × 10^6^ cells/mL) was incubated with the Th1 cell-associated antibodies to CD3 (3 mg/mL), CD8 (5 mg/mL), IFN-γ (5 mg/mL), Th2 cell-associated antibodies to CD3 (3 mg/mL), CD8 (5 mg/mL), IL-4 (5 mg/mL), Th17 cell-associated antibodies to CD3 (5 mg/mL), CD8 (5 mg/mL), IL-17 (1 mg/mL), Treg-associated antibodies to CD4 (1: 100), CD25 (5 mg/mL), CD127 (5 mg/mL), double negative Treg cells (DN CD4^−^CD8^−^ T cells)-associated antibodies to CD3^+^ (2 mg/mL), CD4^−^ (1: 100), CD8^−^ (5 mg/mL), and 10 μL corresponding isotype control antibodies at room temperature for 15 min in the dark. Then the cell suspensions were reacted with 1.5 mL erythrocyte lysis buffer at room temperature for 10 min to lyse the erythrocytes, followed by a 5-min centrifugation at 1500 rpm. The pellet in in each tube was mixed with 1.5 mL PBS for resuspension, followed by re-centrifugation and resuspension. The samples were then detected using a three-color flow cytometer within 2 h, with the cell screening results for the corresponding markers considered to reflect the cell proportion.

### Statistical analysis

Data were analyzed using SPSS 21.0 statistical software (IBM Corp. Armonk, NY, USA). Measurement data were expressed as mean ± standard deviation. Data between two groups were compared using *t*-test when it conforms to the normal distribution, while those among multiple groups were compared by means of one-way analysis of variance. Enumeration data were expressed as either a percentage or rate and examined using a Chi-square test. Pearson correlation analysis was applied to determine the correlation between two variables. *p* <  0.05 was considered to be indicative of significant difference.

## Results

A previous study concluded that similar SLE lesions appeared in VDR-knockout mice. Thus, SLE VDR-insert mice were selected for the purposes of the study to investigate the role of the overexpression of VDR in the alleviation of SLE in mice [21].

### Peripheral blood of SLE patients exhibits reduced hemoglobin and platelets

SLE is predominately associated with immunopathogenesis. As such, altered parameters of peripheral blood are associated with the progression and diagnosis of SLE. Compared to healthy controls, the white blood cell count, red blood cell count and the mean platelet volume (MPV) of incipient SLE patients did not significantly differ, while the hemoglobin as well as the platelet count detected were significantly smaller than in controls (*p* <  0.05; Table 3). The results suggested there to be a lower level of hemoglobin and fewer platelets among SLE patients.

**Table 3:** Comparisons of the parameters of peripheral blood cells between incipient SLE patients and healthy controls

Parameters in the peripheral blood	Incipient SLE patients (*n* = 149)	Healthy controls (*n* = 150)	*p*
White blood cell count/ × 10^9^ L^−1^	5.45 ± 2.28	5.75 ± 1.26	0.159
Red blood cell count/ × 10^12^ L^− 1^	3.96 ± 0.67	4.08 ± 0.39	0.059
Hemoglobin/ (g/L)	107.62 ± 35.82^*^	138.60 ± 10.10	< 0.001
Platelet count/ × 10^9^ L^− 1^	166.34 ± 83.35^*^	201.46 ± 43.46	< 0.001
MPV/fL	8.88 ± 1.95	9.11 ± 0.98	0.198

Notes: Data were analyzed by the independent sample *t*-test; ^*^ *p* < 0.05 compared with the healthy controls; *SLE* systemic lupus erythematosus, *MPV* mean platelet volume

### Treg cell proportion is reduced in incipient SLE patients

**Fig. 1:**
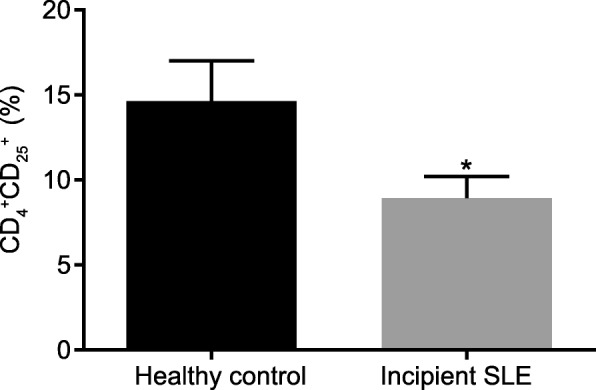
Incipient SLE patients exhibit reduced Treg cell proportion. The data were analyzed using independent sample *t*-test. * *p* < 0.05 vs healthy controls. SLE, systemic lupus erythematosus. Treg, regulatory T cells

CD4^+^ and CD25^+^ cells were isolated from the peripheral blood of incipient SLE patients and healthy controls in order to determine Treg cell proportion. The results obtained indicated that the Treg cell proportion in peripheral blood of incipient SLE patients was reduced when compared with that in peripheral blood of healthy controls (*p* <  0.05; Fig. 1).

### 1,25(OH)_2_D_3_ and VDR are negatively correlated with Skp2 and positively correlated with p27 in SLE patients

**Fig. 2:**
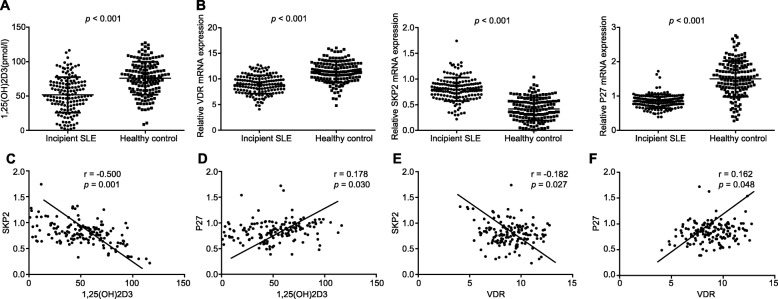
1,25(OH)_2_D_3_ and VDR are negatively correlated with Skp2 and positively correlated with p27 in SLE patients. **a** comparison of the 1,25-(OH)_2_D_3_ content in incipient SLE patients and healthy controls. **b** mRNA expression of VDR, p27 and Skp2. **c** correlation between 1,25(OH)_2_D_3_ and Skp2 expression. **d** correlation between 1,25(OH)_2_D_3_ and p27 expression. **e** correlation of VDR and Skp2 expression. **f** correlation between VDR and p27 expression. *n* = 149 for SLE patients and *n* = 150 for healthy controls; the independent sample *t*-test was performed to analyze data; * *p* < 0.05 vs. the healthy controls; VDR, vitamin D receptor; Skp2, S-phase kinase-associated protein 2; SLE, systemic lupus erythematosus

The expression of 1,25(OH)_2_D_3_, VDR, Skp2 and p27 in renal tubular cells of incipient SLE patients and healthy controls was determined using RT-qPCR. Compared with healthy controls, the Skp2 mRNA expression in the renal tubular cells of SLE patients was significantly higher, while the mRNA expression of 1,25-(OH)_2_D_3_, VDR and p27 was significantly lower (*p* <  0.05; Fig. 2a, b). There was a significant association between SLE and the expression of 1,25-(OH)_2_D_3_ and VDR. The Skp2 expression of patients was relatively elevated, while that of p27 was decreased. The Pearson correlation analysis revealed a significantly negative correlation between the expression of Skp2 and concentrations of 1,25-(OH)_2_D_3_ (r = − 0.500; *p* = 0.001) and VDR (r = − 0.182; *p* = 0.027), and positive correlations between the expression of p27 and the contents of 1,25-(OH)_2_D_3_ (r = 0.178; *p* = 0.030) and VDR (r = 0.162; *p* = 0.048) (Fig. 2c-f). These results indicated that 1,25(OH)_2_D_3_/VDR downregulated Skp2 expression and upregulated p27 expression.

### 1,25(OH)_2_D_3_/VDR alleviates SLE symptoms of SLE mice

**Fig. 3:**
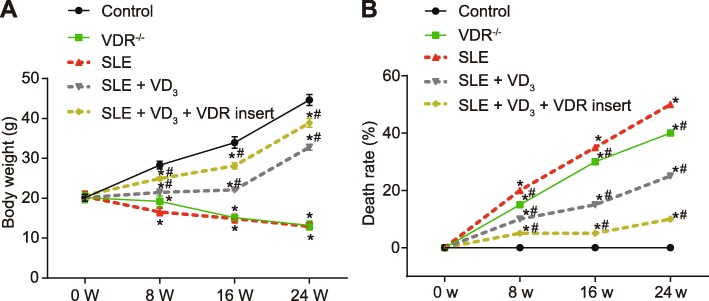
1,25(OH)_2_D_3_/VDR alleviates SLE symptoms of SLE mice. **a** body weight of mice in each group at 0–24 W. **b** death rate of mice in each group at 0–24 W. *n* = 20, two-way analysis of variance was performed to analyze data. * *p* < 0.05 vs. the control group; # *p* < 0.05 vs. the SLE group; SLE, systemic lupus erythematosus; VDR, vitamin D receptor; W, week

The body weight of the mice in the control group progressively increased, and the animals showed no signs of skin ulcers, erosion or urinary system infection. No animals died during the follow-up interval. Compared with the control group, mice in the VDR^−/−^ group displayed symptoms of loose hair, skin ulcers and erosion, urinary systemic infections, and progressive body weight loss (*p* < 0.05). Aggravated skin ulcerations and lower body weights of SLE mice worsened with time compared with mice at 0 W (*p* < 0.05). The symptoms of skin ulceration and erosion, infection and weight loss of mice in the SLE + VD_3_ group were all less pronounced compared to the mice in the SLE group (*p* < 0.05). Mice in the SLE + VD_3_ + VDRinsert group exhibited distinctly improved symptoms in the hair, skin and the urinary tract, exceeding those of the SLE + VD_3_ group. Compared with the SLE group, the weight of the SLE + VD_3_ + VDRinsert mice was significantly greater (*p* < 0.05). There was no significant difference in weight between the SLE + VD_3_ + VDRinsert group and the control group (Fig. 3a-b). Thus, 1,25(OH)2D3/VDR rescued SLE mice from pathology.

### 1,25(OH)_2_D_3_/VDR reduces the contents of BUN and Cr and urinary protein levels in SLE mice

The Coomassie brilliant blue method was used to detect the contents of BUN and Cr and urinary protein levels. At 0 W, the contents of BUN and Cr and urinary protein levels of SLE mice (in the SLE, SLE + VD_3_ and SLE + VD_3_ + VDRinsert groups) were significantly higher than those of control mice (*p* < 0.05), while the mice in the VDR^−/−^ group showed no significant differences in these markers (*p* > 0.05). Compared with 0 W, the contents of BUN, and Cr and urinary protein levels of control mice had not changed by W 24 (*p* > 0.05), while these indexes in mice of the VDR^−/−^ group were significantly increased (*p* < 0.05). These indexes in the SLE mice had slightly increased by W 24, but the markers in the SLE + VD_3_ and SLE + VD_3_ + VDRinsert groups had diminished significantly by W 24 (*p* < 0.05). At 24 W, contents of BUN and Cr and urinary protein levels of mice in the VDR^−/−^ group were significantly elevated relative to baseline (*p* < 0.05). In comparison with the SLE group, the contents of BUN and Cr and urinary protein levels of mice in the SLE + VD_3_ and SLE + VD_3_ + VDRinsert groups were significantly reduced at week 24 (*p* < 0.05) with the contents in the mice in the SLE + VD_3_ + VDRinsert group even decreased to the normal level (Table 4). These results indicated that 1,25(OH)_2_D_3_/VDR reduced the elevations of BUN and Cr and urinary protein levels otherwise seen in SLE mice.

**Table 4:** Comparisons of the blood biochemical indicators of mice in each group

Biochemical indicators	Control (*n* = 10)	VDR^−/−^ (n = 10)	SLE (*n* = 10)	SLE + VD_3_ (*n* = 10)	SLE + VD_3_ + VDRinsert (n = 10)
BUN					
0 W	5.32 ± 0.71	5.48 ± 0.25	13.03 ± 1.60^*^	12.76 ± 0.54^*^	12.22 ± 0.40^*^
24 W	5.26 ± 0.54	9.53 ± 0.37^*$^	13.76 ± 1.12^*^	6.95 ± 0.65^#$*^	5.72 ± 0.58^#$^
Cr					
0 W	22.24 ± 2.54	22.80 ± 1.06	35.87 ± 1.15^*^	35.38 ± 1.22^*^	34.62 ± 1.27^*^
24 W	20.93 ± 1.08	30.43 ± 1.46^$^	37.09 ± 1.59^*^	25.06 ± 1.51^#$*^	22.04 ± 0.67^#$^
Urine protein (μg/mL)					
0 W	45 ± 10	43 ± 6	86 ± 11^*^	88 ± 15^*^	86 ± 17^*^
24 W	48 ± 11	77 ± 12^*$^	91 ± 14^*^	62 ± 14^*#$^	57 ± 13^*#$^

Notes: one-way analysis of variance was performed to analyze data; ^*^ *p* < 0.05 compared with the control group; ^*#*^ *p* < 0.05 compared with the SLE group; the paired *t*-test was performed to analyze data; ^$^ *p* < 0.05 compared with the 0 W; *SLE* systemic lupus erythematosus, *VDR* vitamin D receptor; *BUN* blood urea nitrogen, *Cr* creatinine

### 1,25(OH)_2_D_3_/VDR reduces the degree of renal damage and inflammatory cell infiltration in SLE mice

**Fig. 4:**
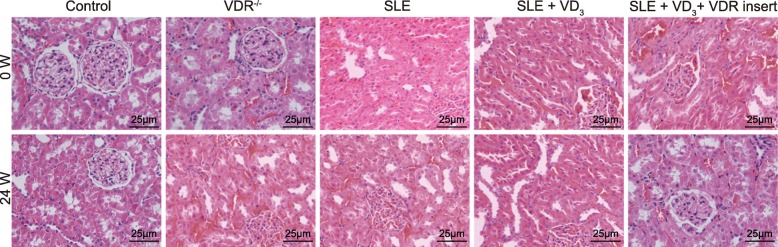
HE staining mouse renal tissues reflecting the effect of 1,25(OH)_2_D_3_/VDR on pathological changes (× 400). HE staining of renal tissues in each group; SLE, systemic lupus erythematosus; HE, hematoxylin and eosin; VDR, vitamin D receptor

**Fig. 5:**
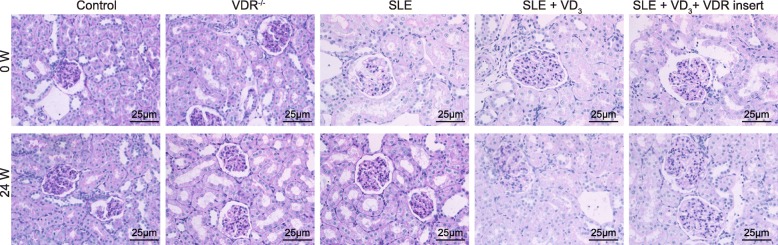
PAS staining of mouse renal tissues reflecting the effect of 1,25(OH)_2_D_3_/VDR on pathological changes of renal tissues of SLE mice (× 400). SLE, systemic lupus erythematosus; PAS, periodic acid Schiff; VDR, vitamin D receptor

HE staining and PAS staining illustrated that the morphologies of the renal tissues in the control group and VDR^−/−^ group were normal at 0 W, while the renal tissues of SLE mice (in the SLE, SLE + VD_3,_ and SLE + VD_3_ + VDRinsert groups) exhibited signs of damage, i.e. increased glomerular volume, deposition of fuchsinophilic proteins in the mesangial area and subendothelial area, infiltration of chronic inflammatory cells in the glomerulus and renal interstitium, with signs of monocyte infiltration of the glomerular capillary. After 24-weeks, the mice in the control group exhibited no distinct signs of deterioration, while the degree of renal damage and infiltration of the inflammatory cells of the normal mice in the VDR^−/−^ group were markedly elevated compared with the control group. The degree of SLE symptoms of the mice in the SLE group was more severe, while the degree of SLE symptoms of mice in the SLE + VD_3_ and SLE + VD_3_ + VDRinsert groups were attenuated, showing less mesangial cell mild hyperplasia, slight leukocyte infiltration, and only a small amount of fuchsinophilic protein deposition (Figs. 4 and 5). Thus, treatment with 1,25(OH)_2_D_3_/VDR rescued SLE mice from renal damage and inflammatory cell infiltration.

### 1,25(OH)_2_D_3_/VDR reduces the precipitation intensity of the immune complex (IgG, IgA, IgM) and complement (C_3_, C_1q_) in SLE mice

**Fig. 6:**
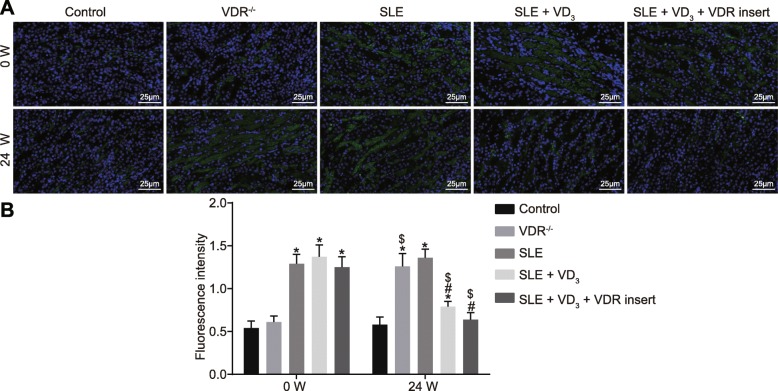
1,25(OH)_2_D_3_/VDR diminishes the precipitation intensity of the immune complex IgG in SLE mice. **a** the immunofluorescence staining (× 400) of IgG. **b** the quantitative analysis of fluorescence intensity of IgG in each group. *n* = 20, one-way analysis of variance was used to analyze data; * *p* < 0.05 vs. the control group; # *p* < 0.05 vs. the SLE group; paired *t*-test was performed to analyze data; $ *p* < 0.05 vs. 0 W; SLE, systemic lupus erythematosus; VDR, vitamin D receptor; W, week; IgG, immunoglobulin G

**Fig. 7:**
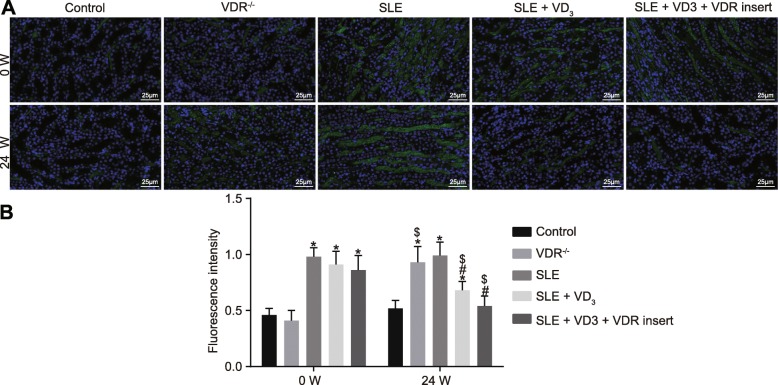
1,25(OH)_2_D_3_/VDR reduces the precipitation intensity of the immune complex IgA in SLE mice. **a** the immunofluorescence staining (× 400) of IgA. **b** the quantitative analysis of fluorescence intensity of IgA in each group. *n* = 20, one-way analysis of variance was used to analyze data; * *p* < 0.05 vs. the control group; # *p* < 0.05 vs. the SLE group; paired *t*-test was performed to analyze data; $ *p* < 0.05 vs. 0 W; SLE, systemic lupus erythematosus; VDR, vitamin D receptor; W, week; IgA, immunoglobulin A

**Fig. 8:**
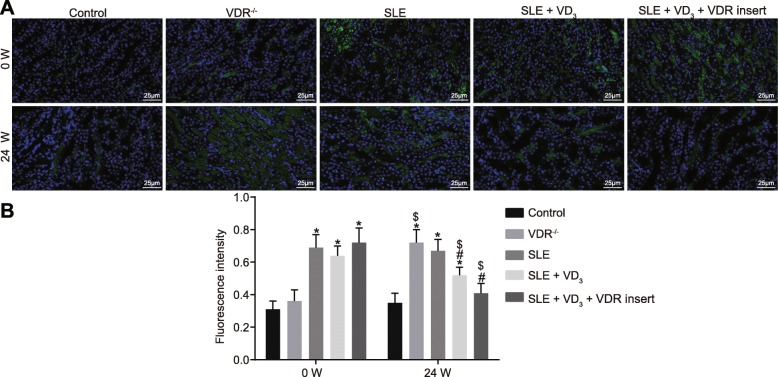
1,25(OH)_2_D_3_/VDR reduces the precipitation intensity of the immune complex IgM in SLE mice. **a** the immunofluorescence staining (× 400) of IgM. **b** the quantitative analysis of fluorescence intensity of IgM in each group. n = 20, one-way analysis of variance was applied to analyze the data; * *p* < 0.05 vs. the control group; # *p* < 0.05 vs. the SLE group; paired *t*-test was performed to analyze data; $ *p* < 0.05 vs. 0 W; SLE, systemic lupus erythematosus; VDR, vitamin D receptor; W, week; IgM, immunoglobulin M

**Fig. 9:**
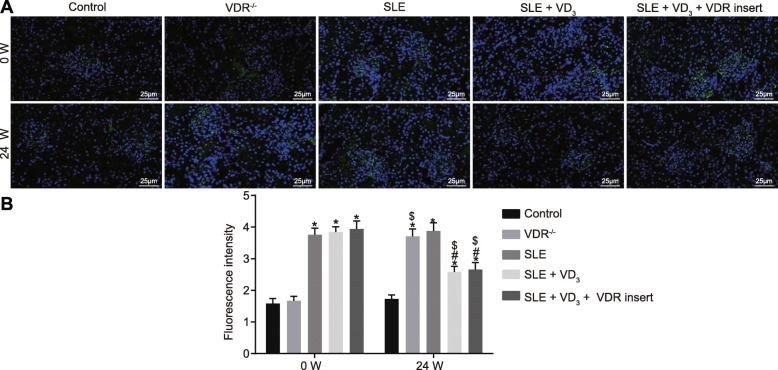
1,25(OH)_2_D_3_/VDR reduces the precipitation intensity of the complement C_3_ in SLE mice. **a** the immunofluorescence staining (× 400) of C_3_. **b** the quantitative analysis of fluorescence intensity of C_3_ in each group. n = 20, one-way analysis of variance was used to analyze data; * *p* < 0.05 vs. the control group; # *p* < 0.05 vs. the SLE group; paired *t*-test was performed to analyze data; $ *p* < 0.05 vs. 0 W; SLE, systemic lupus erythematosus; VDR, vitamin D receptor; W, week

**Fig. 10:**
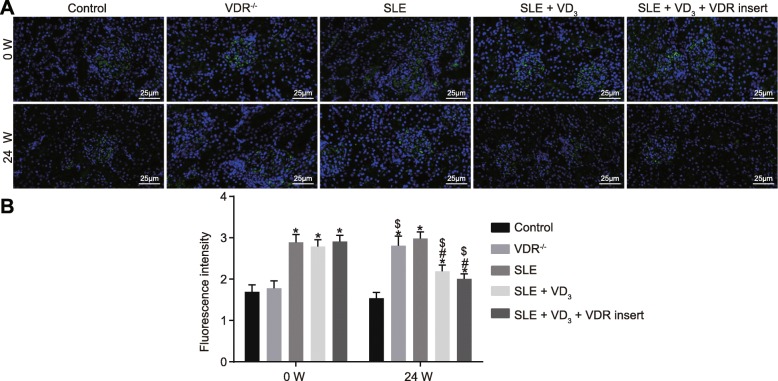
1,25(OH)_2_D_3_/VDR reduces the precipitation intensity of the complement C_1q_ in SLE mice. **a** the immunofluorescence staining (× 400) of C_1q_. **b** the quantitative analysis of fluorescence intensity of C_1q_ in each group. n = 20, one-way analysis of variance was used to analyze data; * *p* < 0.05 vs. the control group; # *p* < 0.05 vs. the SLE group; paired *t*-test was performed to analyze data; $ *p* < 0.05 vs. 0 W; SLE, systemic lupus erythematosus; VDR, vitamin D receptor; W, week

Immunopathological changes and the precipitation intensity of the immune complex of mice in each group were observed via immunofluorescence staining. At 0 W, compared with the control and VDR^−/−^ groups, the intensity of immune proteins and complement depositions of SLE mice displayed an obvious “full house” phenomenon, characterized by positive staining for IgG, IgA, IgM, C_3_, and C_1q_. At week 24, the deposition intensity of the immune complex (IgG, IgA, IgM) and complement (C_3_, C_1q_) of mice in the control group had not changed significantly (*p* > 0.05). The deposition intensity of these immunological markers of mice in the VDR^−/−^ group was significantly increased at week 24 relative to control mice (*p* < 0.05). Compared with the SLE group, the deposition intensity of the immunological markers in mice of the SLE + VD_3_ and SLE + VD_3_ + VDRinsert groups had reduced (*p* < 0.05), while mice in the SLE + VD_3_ + VDRinsert group showed no significant difference in intensity compared with those in the control group (Figs. 6, 7, 8, 9 and 10). Thus, 1,25(OH)_2_D_3_/VDR decreased the precipitation intensity of the immune complex (IgG, IgA, IgM) and complement (C_3_, C_1q_) in SLE mice.

### 1,25(OH)_2_D_3_/VDR downregulates the levels of IL-4, IL-10, IL-17 and INF-γ in SLE mice

ELISA was performed in order to determine the levels of the inflammatory factors (IL-4, IL-10, IL-17, and INF-γ). The results obtained indicated that at 0 W, compared with mice in the control and VDR^−/−^ groups, the IL-4, IL-10, IL-17 and INF-γ levels of SLE mice (in the SLE, SLE + VD_3_, and SLE + VD_3_ + VDRinsert groups) were significantly increased (*p* < 0.05). At the 24th week, the levels of these factors in mice in the control group had not changed significantly (*p* > 0.05). Compared with the control group, the levels of these factors in mice in the VDR^−/−^ group were significantly elevated (*p* < 0.05). Compared with the SLE group, the levels of these factors in mice in the SLE + VD_3_ and SLE + VD_3_ + VDRinsert groups were significantly lower (*p* < 0.05). By the 24th week, there was no significant difference identified in the IL-4, IL-10, IL-17 and INF-γ levels between the SLE + VD_3_ + VDRinsert and control groups (Table 5). These results suggested that 1,25(OH)_2_D_3_/VDR downregulated the levels of inflammatory factors (IL-4, IL-10, IL-17, and INF-γ) in SLE mice.

**Table 5:** Comparisons of inflammatory factors (IL-4, IL-10, IL-17 and INF-γ) of mice in each group (pg/mL)

Group	IL-4		IL-10		IL-17		INF-γ	
	0 W (n = 10)	24 W (n = 10)	0 W (n = 10)	24 W (n = 10)	0 W (n = 10)	24 W (n = 10)	0 W (n = 10)	24 W (n = 10)
Control	9.24 ± 3.0	9.52 ± 2.01	30.12 ± 7.58	34.98 ± 8.14	8.73 ± 2.41	7.51 ± 2.50	11.93 ± 0.78	11.33 ± 1.37
VDR^−/−^	9.70 ± 3.14	20.55 ± 4.18^*$^	36.12 ± 7.04	79.39 ± 7.30^*$^	8.45 ± 1.47	13.95 ± 2.68^*$^	12.29 ± 4.77	23.65 ± 5.12^*$^
SLE	35.80 ± 6.02^*^	38.39 ± 2.56^*^	82.08 ± 13.35^*^	90.44 ± 10.83^*^	15.76 ± 3.83^*^	16.27 ± 3.10^*^	29.92 ± 7.81^*^	32.67 ± 4.58
SLE + VD_3_	34.71 ± 3.64^*^	12.27 ± 3.55^#$^	83.27 ± 11.44^*^	43.74 ± 4.83^#$^	15.60 ± 2.96^*^	9.77 ± 5.28^#$^	28.71 ± 5.10^*^	14.94 ± 3.13^*#$^
SLE + VD_3_ + VDRinsert	34.14 ± 3.11^*^	11.79 ± 1.84^#$^	81.59 ± 9.20^*^	41.69 ± 5.18^#$^	15.02 ± 3.62^*^	8.40 ± 2.02^#$^	28.62 ± 2.72^*^	14.32 ± 2.11^#$^

Notes: one-way analysis of variance was performed to analyze data; ^*^ *p* < 0.05 compared with the healthy controls; ^#^ *p* < 0.05 compared with the SLE group; the paired *t*-test was performed to analyze data; ^$^ *p* < 0.05 compared with the 0 W; *SLE* systemic lupus erythematosus, *IL* Interleukin, *INF* Interferon, *VDR* vitamin D receptor

### 1,25(OH)_2_D_3_/VDR decreases the levels of anti-nRNP IgG and anti-dsDNA IgG in SLE mice

ELISA was applied in order to detect the levels of anti-nRNP IgG and anti-dsDNA IgG in SLE mice. Based on the ELISA results, before treatment (0 W), the levels of anti-nRNP IgG and anti-dsDNA IgG in SLE mice (in the SLE, SLE + VD_3_, SLE + VD_3_ + VDRinsert groups) were profoundly upregulated compared with the levels in the control and VDR^−/−^ groups (*p* < 0.05). With 24 W treatment, the levels of anti-nRNP IgG and anti-dsDNA IgG in the control group displayed no significant difference relative to baseline (*p* > 0.05), while the levels of anti-nRNP IgG and anti-dsDNA IgG in the VDR^−/−^ group were elevated when compared with those in the control group (*p* < 0.05). Compared with the SLE group, the levels of anti-nRNP IgG and anti-dsDNA IgG were significantly downregulated in the SLE + VD_3_ and SLE + VD_3_ + VDRinsert groups after treatment (*p* < 0.05), while the SLE + VD_3_ + VDRinsert group displayed no significant difference in the antibody levels versus control mice at 24 W (Table 6). These findings demonstrated that 1,25(OH)_2_D_3_/VDR decreased the levels of anti-nRNP IgG and anti-dsDNA IgG in SLE mice.

**Table 6:** Comparison of the anti-nRNP IgG and anti-dsDNA IgG levels of mice in each group

Group	Anti-nRNP IgG		Anti-dsDNA IgG	
	0 W (n = 10)	24 W (n = 10)	0 W (n = 10)	24 W (n = 10)
Control	0.51 ± 0.09	0.62 ± 0.06	0.12 ± 0.04	0.15 ± 0.05
VDR^−/−^	0.56 ± 0.08	1.13 ± 0.16^*$^	0.16 ± 0.01	0.45 ± 0.10^*$^
SLE	1.12 ± 0.17^*^	1.25 ± 0.13^*^	0.61 ± 0.07^*^	0.52 ± 0.11^*^
SLE + VD_3_	1.19 ± 0.05^*^	0.81 ± 0.21^#$^	0.63 ± 0.12^*^	0.28 ± 0.02^#$^
SLE + VD_3_ + VDRinsert	1.23 ± 0.12^*^	0.65 ± 0.15^#$^	0.69 ± 0.14^*^	0.18 ± 0.03^#$^

Notes: one-way analysis of variance was performed to analyze data; ^*^ *p* < 0.05 compared with the control group; ^*#*^ *p* < 0.05 compared with the SLE group; the paired *t*-test was performed to analyze data; ^$^ *p* < 0.05 compared with the 0 W; *SLE* systemic lupus erythematosus, *IgG* immunoglobulin G; *VDR* vitamin D receptor

### 1,25(OH)_2_D_3_/VDR regulates splenic immune cells in SLE mice

**Fig. 11:**
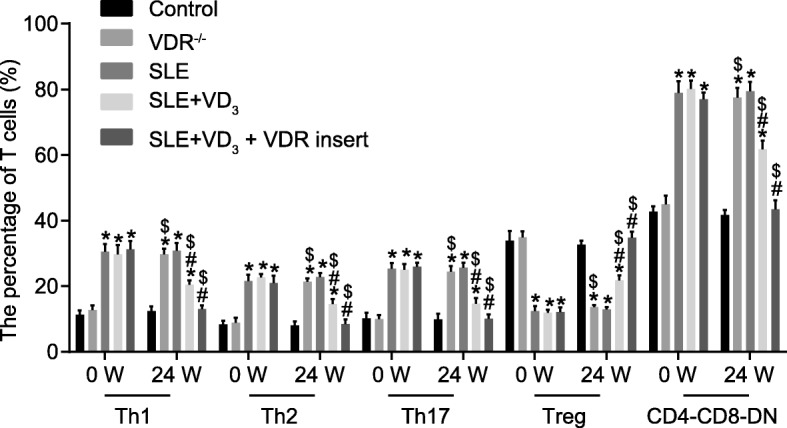
1,25(OH)_2_D_3_/VDR regulates the distribution of splenic immune cells (Treg, Th17, Th1, Th2, and CD4-CD8-DN) in SLE mice (%). n = 20, one-way analysis of variance was used to analyze data; * *p* < 0.05 vs. the control group; # *p* < 0.05 vs. the SLE group; *t*-test was conducted; $ *p* < 0.05 vs. 0 W; SLE, systemic lupus erythematosus; VDR, vitamin D receptor; W, week; Treg, regulatory T; Th17, T helper 17; Th1, T helper 1; Th2, helper 2

Flow cytometry analysis showed that the numbers of T helper cells (Th17, Th1, Th2) and CD4^−^CD8^−^ DN cells were significantly increased in the SLE mice (in the SLE, SLE + VD_3_, SLE + VD_3_ + VDRinsert groups) when compared with numbers in the control and VDR^−/−^ groups, while the number of Tregs was reduced (*p* < 0.05). The numbers of Treg, Th17, Th1, Th2, and CD4^−^CD8^−^ DN cells exhibited no significant difference in the control group at the 24 W (all *p* > 0.05), while the VDR^−/−^ group displayed increased numbers of Th17, Th1, Th2, and CD4^−^CD8^−^ DN cells compared with the control group (*p* < 0.05). In comparison to the SLE group, the numbers of Th17, Th1, Th2, and CD4^−^CD8^−^ DN cells were significantly decreased in the SLE + VD_3_ and SLE + VD_3_ + VDRinsert groups after treatment, while the number of Tregs was enhanced (*p* < 0.05), with no significant difference detected in relation to the numbers of Treg, Th17, Th1, Th2, and CD4^−^CD8^−^ DN cells in the SLE + VD_3_ + VDRinsert group relative to control mice at 24 W (Fig. 11). These results reveal that 1,25(OH)_2_D_3_/VDR mediates the number of splenic immune cells in SLE mice.

### 1,25-(OH)_2_D_3_/VDR downregulates the Skp2 expression and upregulates the p27 expression

**Fig. 12:**
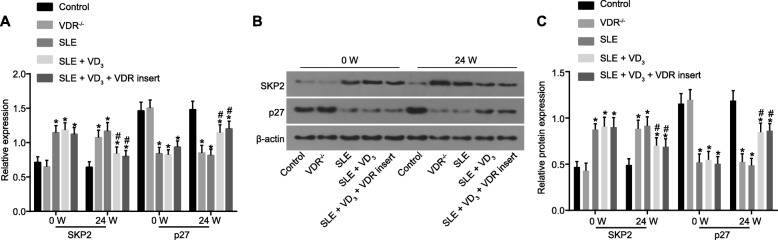
1,25-(OH)_2_D_3_/VDR downregulates the Skp2 expression and upregulates the p27 expression in SLE mice. **a** the mRNA expression of Skp2 and p27 determined by RT-qPCR. **b** the protein expression of Skp2 and p27 measured by western blot analysis. **c** the statistical analysis of panel B; n = 20, one-way analysis of variance was used to analyze data; * *p* < 0.05 vs. the control group; # *p* < 0.05 vs. the SLE group; Skp2, S-phase kinase-associated protein 2; SLE, systemic lupus erythematosus; RT-qPCR, reverse transcription quantitative polymerase chain reaction

RT-qPCR and western blot analysis that before treatment (0 W), the mRNA expression of Skp2 in SLE mice (in the SLE, SLE + VD_3_ and SLE + VD_3_ + VDRinsert groups) was significantly increased, while the expression of p27 decreased compared with expression in control mice (*p* < 0.05). Mice in the VDR^−/−^ group displayed no significant difference in mRNA expression of Skp2 and p27 (*p* > 0.05). After 24-W of treatment, the mRNA expression of Skp2 and p27 in the control and SLE groups did not differ (*p* > 0.05). The mRNA expression of Skp2 in the VDR^−/−^ group was notably elevated while that of p27 was markedly decreased (*p* < 0.05), with an opposite trend identified in the SLE + VD_3_ and SLE + VD_3_ + VDRinsert groups (*p* < 0.05). Compared with the control group, the mRNA expression of Skp2 in the VDR^−/−^ group displayed notable increases, while that of p27 was significantly decreased (*p* < 0.05). Compared with the SLE group, the mRNA expression of Skp2 in the SLE + VD_3_ and SLE + VD_3_ + VDRinsert groups was significantly decreased, while that of p27 was significantly increased, with the changes in the SLE + VD_3_ + VDRinsert group being more significant (*p* < 0.05; Fig. 12a). Compared with the control group, the relative protein expression of p-Skp2 in the VDR^−/−^ group was significantly increased after 24 W of treatment, while that of p27 was significantly decreased (*p* < 0.05). Compared with the SLE group, the relative protein expression of Skp2 in the SLE + VD_3_ and SLE + VD_3_ + VDRinsert groups was significantly decreased, while that of p27 was significantly increased (*p* < 0.05; Fig. 12b, c). The results demonstrated that 1,25-(OH)_2_D_3_/VDR downregulated the expression of Skp2 in addition to upregulated expression of p27.

## Discussion

Accumulating evidence has elucidated a close correlation between 1,25-(OH)_2_D_3_/VDR and SLE [22, 23]. Moreover, Skp2 and p27 have been shown to function in autoimmune disease development [12, 13]. In the present study, our aim was to investigate the effects of 1,25-(OH)_2_D_3_/VDR treatment in SLE that are obtained by regulation the Skp2/p27 signaling pathway, with the hope of developing a novel therapeutic target for the treatment of SLE. Our results demonstrated that 1,25-(OH)_2_D_3_/VDR promotes the recovery of SLE in mice by downregulating Skp2 and upregulating p27 expression.

First, we found that the hemoglobin and platelet count in patients with incipient SLE were significantly decreased compared with those in healthy controls. A previous study has revealed that patients with SLE exhibit markedly decreased platelet counts [24]. Importantly, the present study found that the expression of Skp2 in the incipient SLE patients was notably increased, while the expression of 1,25-(OH)_2_D_3_, VDR and p27 was markedly decreased, highlighting the relationship between SLE and 1,25-(OH)_2_D_3_/VDR. Moreover, the variant of VDR may negatively influence the clinical presentations in the process of SLE [7]. Furthermore, VDR led to the reduced production of many interleukins and IFN-γ, and dysregulation of T cells contributed to an excessive production of antibodies acting against DNA and self-proteins [25]. Furthermore, 1,25-(OH)_2_D_3_ regulates the expression of various apoptosis factors which directly induce apoptosis via caspase activation, demonstrating its ability to interfere with the immunostimulatory effects of SLE [26]. A key observation of our study was that 1,25(OH)_2_D_3_/VDR regulated splenic immune cells in SLE mice. Tregs, including induced Tregs and naturally arising Foxp3^+^ nTregs, together play vital roles in immune escape and immunotherapy failure in cancer patients [27]. Therefore, maintaining the appropriate quantity of Tregs is vitally important in immunological fitness. However, Skp2 is a critical functional and molecular switch for Tregs, and excessive expression of Skp2 in Foxp3^+^ nTregs weakens the expression of Foxp3 and subsequently attenuates its suppressive function [12].

1,25-(OH)_2_D_3_/VDR was found to downregulated the expression of Skp2 and upregulated the expression of p27 in SLE patients. Iglesias et al. concluded that p27, as a cell cycle inhibitor, pays a crucial role in regulating Tregs in addition to demonstrating an ability to enhance the activity and differentiation of Tregs as a positive regulator of the signaling pathway in CD4+ cells [13]. The Pearson correlation analysis highlighted a correlation between Skp2 and 1,25-(OH)_2_D_3_/VDR, suggesting them to be negatively related, while a positive association between p27 and 1,25-(OH)_2_D_3_/VDR was detected. Yang et al., revealed diminished Skp2 protein levels in cells in response to 1,25-(OH)_2_D_3_ after 24 and 48 h of treatment [15]. In addition, 1,25(OH)_2_D_3_ analogs have been shown to act via two cell type-dependent mechanisms to elevate the expression of p27 and decrease the expression of Skp2 [16]. The deteriorated symptoms of mice without VDRinsert also further underlined the vital significance of VDR in SLE, highlighting the potential of 1,25-(OH)_2_D_3_/VDR as a promising treatment for SLE.

1,25(OH)_2_D_3_/VDR downregulates the levels of IL-4, IL-10, IL-17 and INF-γ in SLE mice. It has been indicated that the cytokine production in SLE is promoted by the alterations in Th1 and Th2, which may exacerbate the condition of patients suffering from the condition [28]. Cytokines formed by Th17 cells, such as IL-17, play crucial roles in the process of inflammation which can result in tissue damage, with upregulated levels of IL-17 observed in patients with a variety of autoimmune diseases, including SLE [22]. The activity of these cytokines can trigger a chain of inflammatory responses, leading to autoantibody production by B cells in SLE [29]. Hence, when compared with the control and VDR^−/−^ mice, the inflammatory factors IL-4, IL-10, IL-17 and INF-γ were all significantly elevated. However, other evidence has suggested that vitamin D can inhibit the expansion of T cells and modulate the expression of cytokines with a Th2 bias [28], which in the present context resulted in a decrease in the inflammatory cytokines in the SLE + VD_3_ and SLE + VD_3_ + VDRinsert groups. Other observations revealed continually increased expression of inflammatory factors in the VDR^−/−^ group, which ultimately emphasized the efficacy of VDR in SLE.

## Conclusion

Taken together, the key evidence obtained during the current study suggests that 1,25(OH)_2_D_3_/VDR acts to stimulate the recovery from SLE symptoms by downregulating Skp2 and upregulating the p27, highlighting a promising novel therapeutic target for future SLE treatment.

## Supplementary information

**Additional file 1: Figure S1.** Construction of targeting vector for VDR ablation. A schematic representation of the VDR gene is displayed on the basis of the structure of the human gene and characterization of the sequences from exon 3 to exon 9 of the mouse gene. The exons are numbered and showed by solid boxes. A partial restriction map is demonstrated for the following enzymes: R, EcoRI; X, XbaI; S, SacI. The XbaI site indicated by the asterisk was derived from the phage arm. A 5-kb XbaI fragment 59 to exon 3 and a 3.5-kb XbaI fragment 39 to exon 3 of the mouse VDR gene were applied as the targeting sequences. The SacI-XbaI fragment applied as a probe for identifying homologous recombinants is showed as well.

## Data Availability

The datasets used and/or analyzed during the current study are available from the corresponding author on reasonable request.

## Abbreviations

BSA: Bovine serum albumin
BUN: Blood urea nitrogen
ELISA: Enzyme-linked immunosorbent assay
FITC: Fluorescein isothiocyanate
HE: Hematoxylin and Eosin
MPV: Mean platelet volume
PAS: Periodic acid Schiff
PBMC: Peripheral blood mononuclear cells
PBS: Phosphate buffer solution
PVDF: Polyvinylidene fluoride
RT-qPCR: Reverse transcription quantitative polymerase chain reaction
SLE: Systemic lupus erythematosus
SPF: Specific pathogen free
TBST: Tris-buffered saline Tween-20
VDR: Vitamin D receptor

## References

[1] Conti F, Ceccarelli F, Massaro L, Pacucci VA, Miranda F, Truglia S (2013). Evaluation of the patient acceptable symptom state (PASS) in Italian patients affected by systemic lupus erythematosus: association with disease activity indices. PLoS One.

[2] Shelton KA, Cline JM, Cann JA (2013). 17-beta estradiol reduces atherosclerosis without exacerbating lupus in ovariectomized systemic lupus erythematosus-susceptible LDLr(−/−) mice. Atherosclerosis.

[3] Tasdemir M, Hasan C, Agbas A, Kasapcopur O, Canpolat N, Sever L (2016). Sjogren's syndrome associated with systemic lupus erythematosus. Turk Pediatri Ars.

[4] He XJ, Ding Y, Xiang W, Dang XQ (2016). Roles of 1,25(OH)2D3 and vitamin D receptor in the pathogenesis of rheumatoid arthritis and systemic lupus Erythematosus by regulating the activation of CD4+ T cells and the PKCdelta/ERK signaling pathway. Cell Physiol Biochem.

[5] Santos EC, Pinto AC, Klumb EM, Macedo JM (2016). Polymorphisms in NAT2 (N-acetyltransferase 2) gene in patients with systemic lupus erythematosus. Rev Bras Reumatol Engl Ed.

[6] Han YS, Min Yang C, Lee SH, Shin JH, Moon SW, Kang JH (2015). Secondary angle closure glaucoma by lupus choroidopathy as an initial presentation of systemic lupus erythematosus: a case report. BMC Ophthalmol.

[7] Mostowska A, Lianeri M, Wudarski M, Olesinska M, Jagodzinski PP (2013). Vitamin D receptor gene BsmI, FokI, ApaI and TaqI polymorphisms and the risk of systemic lupus erythematosus. Mol Biol Rep.

[8] Bogaczewicz J, Sysa-Jedrzejowska A, Arkuszewska C, Zabek J, Kontny E, McCauliffe D (2012). Vitamin D status in systemic lupus erythematosus patients and its association with selected clinical and laboratory parameters. Lupus..

[9] Sun X, Su S, Chen C, Han F, Zhao C, Xiao W (2014). Long-term outcomes of intensity-modulated radiotherapy for 868 patients with nasopharyngeal carcinoma: an analysis of survival and treatment toxicities. Radiother Oncol.

[10] Lippens C, Duraes FV, Dubrot J, Brighouse D, Lacroix M, Irla M (2016). IDO-orchestrated crosstalk between pDCs and Tregs inhibits autoimmunity. J Autoimmun.

[11] Ohl K, Tenbrock K (2015). Regulatory T cells in systemic lupus erythematosus. Eur J Immunol.

[12] Wang D, Qin H, Du W, Shen YW, Lee WH, Riggs AD (2012). Inhibition of S-phase kinase-associated protein 2 (Skp2) reprograms and converts diabetogenic T cells to Foxp3+ regulatory T cells. Proc Natl Acad Sci U S A.

[13] Iglesias M, Postigo J, Santiuste I, Gonzalez J, Buelta L, Tamayo E (2013). p27(Kip1) inhibits systemic autoimmunity through the control of Treg cell activity and differentiation. Arthritis Rheum.

[14] Wei Z, Jiang X, Qiao H, Zhai B, Zhang L, Zhang Q (2013). STAT3 interacts with Skp2/p27/p21 pathway to regulate the motility and invasion of gastric cancer cells. Cell Signal.

[15] Yang ES, Burnstein KL (2003). Vitamin D inhibits G1 to S progression in LNCaP prostate cancer cells through p27Kip1 stabilization and Cdk2 mislocalization to the cytoplasm. J Biol Chem.

[16] Lin R, Wang TT, Miller WH, White JH (2003). Inhibition of F-box protein p45(SKP2) expression and stabilization of cyclin-dependent kinase inhibitor p27(KIP1) in vitamin D analog-treated cancer cells. Endocrinology.

[17] Prabha R, Mathew BS, Jeyaseelan V, Kumar TS, Agarwal I, Fleming DH (2016). Development and validation of limited sampling strategy equation for mycophenolate mofetil in children with systemic lupus erythematosus. Indian J Nephrol.

[18] Tedeschi SK, Massarotti E, Guan H, Fine A, Bermas BL, Costenbader KH (2015). Specific systemic lupus erythematosus disease manifestations in the six months prior to conception are associated with similar disease manifestations during pregnancy. Lupus..

[19] Yazdany J, Robbins M, Schmajuk G, Desai S, Lacaille D, Neogi T (2016). Development of the American College of Rheumatology's rheumatoid arthritis electronic clinical quality measures. Arthritis Care Res (Hoboken).

[20] Tuo YL, Li XM, Luo J (2015). Long noncoding RNA UCA1 modulates breast cancer cell growth and apoptosis through decreasing tumor suppressive miR-143. Eur Rev Med Pharmacol Sci.

[21] Ding Y, Liao W, He XJ, Xiang W (2017). Effects of 1,25(OH)2 D3 and vitamin D receptor on peripheral CD4(+) /CD8(+) double-positive T lymphocytes in a mouse model of systemic lupus erythematosus. J Cell Mol Med.

[22] Wahono CS, Rusmini H, Soelistyoningsih D, Hakim R, Handono K, Endharti AT (2014). Effects of 1,25(OH)2D3 in immune response regulation of systemic lupus erithematosus (SLE) patient with hypovitamin D. Int J Clin Exp Med.

[23] Handono K, Sidarta YO, Pradana BA, Nugroho RA, Hartono IA, Kalim H (2014). Vitamin D prevents endothelial damage induced by increased neutrophil extracellular traps formation in patients with systemic lupus erythematosus. Acta Med Indones.

[24] Pundole X, Konoplev S, Oo TH, Lu H (2015). Autoimmune myelofibrosis and systemic lupus erythematosus in a middle-aged male presenting only with severe anemia: a case report. Medicine (Baltimore).

[25] Bradley SJ, Suarez-Fueyo A, Moss DR, Kyttaris VC, Tsokos GC (2015). T cell Transcriptomes describe patient subtypes in systemic lupus Erythematosus. PLoS One.

[26] Lee L, Cui JZ, Cua M, Esfandiarei M, Sheng X, Chui WA (2016). Aortic and cardiac structure and function using high-resolution echocardiography and optical coherence tomography in a mouse model of Marfan syndrome. PLoS One.

[27] Zhuo C, Xu Y, Ying M, Li Q, Huang L, Li D (2015). FOXP3+ Tregs: heterogeneous phenotypes and conflicting impacts on survival outcomes in patients with colorectal cancer. Immunol Res.

[28] Csiszar A, Nagy G, Gergely P, Pozsonyi T, Pocsik E (2000). Increased interferon-gamma (IFN-gamma), IL-10 and decreased IL-4 mRNA expression in peripheral blood mononuclear cells (PBMC) from patients with systemic lupus erythematosus (SLE). Clin Exp Immunol.

[29] Coleman LA, Mishina M, Thompson M, Spencer SM, Reber AJ, Davis WG (2016). Age, serum 25-hydroxyvitamin D and vitamin D receptor (VDR) expression and function in peripheral blood mononuclear cells. Oncotarget..

[30] Orlans FB (1997). Ethical decision making about animal experiments. Ethics Behav.

