# Supplementary material for: 1,25-(OH)2D3/Vitamin D receptor alleviates systemic lupus erythematosus by downregulating Skp2 and upregulating p27
Source: Cell Commun Signal. 2019 Dec 10;17:163. doi: 10.1186/s12964-019-0488-2 (PMC6905035; doi:10.1186/s12964-019-0488-2)
Supplement: Supplementary file 1 — Additional file 1: Figure S1. Construction of targeting vector for VDR ablation. A schematic representation of the VDR gene is displayed on the basis of the structure of the human gene and characterization of the sequences from exon 3 to exon 9 of the mouse gene. The exons are numbered and showed by solid boxes. A partial restriction map is demonstrated for the following enzymes: R, EcoRI; X, XbaI; S, SacI. The XbaI site indicated by the asterisk was derived from the phage arm. A 5-kb XbaI fragment 59 to exon 3 and a 3.5-kb XbaI fragment 39 to exon 3 of the mouse VDR gene were applied as the targeting sequences. The SacI-XbaI fragment applied as a probe for identifying homologous recombinants is showed as well. [file 12964_2019_488_MOESM1_ESM.docx]

**Figure S1.** Construction of targeting vector for VDR ablation. A schematic representation of the VDR gene is displayed on the basis of the structure of the human gene and characterization of the sequences from exon 3 to exon 9 of the mouse gene. The exons are numbered and showed by solid boxes. A partial restriction map is demonstrated for the following enzymes: R, EcoRI; X, XbaI; S, SacI. The XbaI site indicated by the asterisk was derived from the phage arm. A 5-kb XbaI fragment 59 to exon 3 and a 3.5-kb XbaI fragment 39 to exon 3 of the mouse VDR gene were applied as the targeting sequences. The SacI-XbaI fragment applied as a probe for identifying homologous recombinants is showed as well.


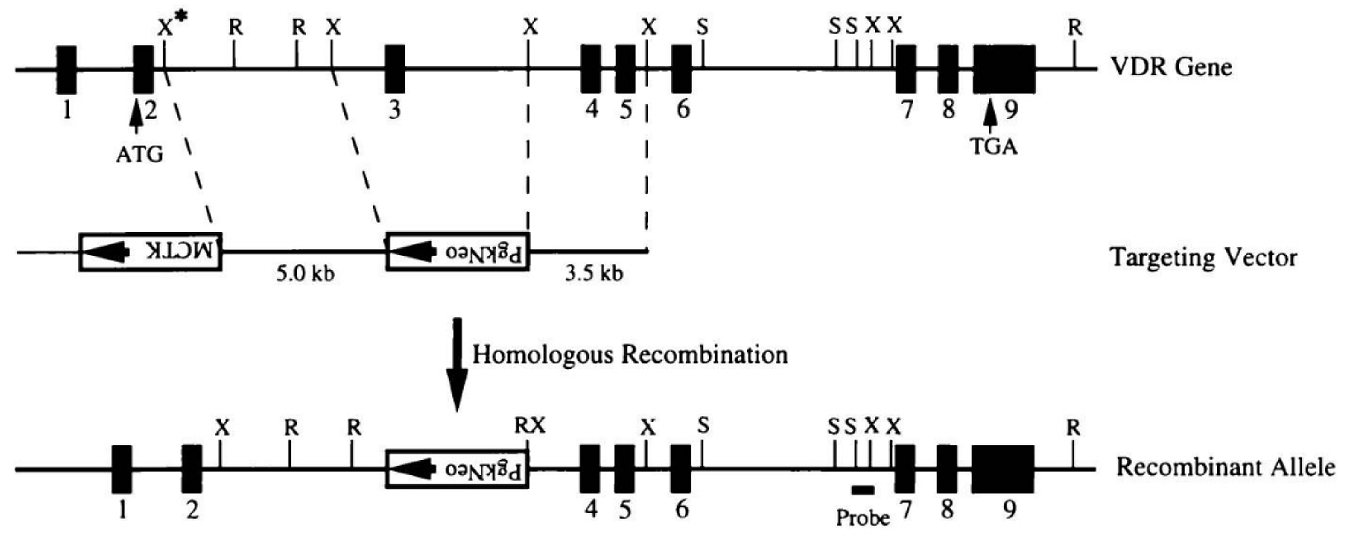


**Figure S1.** Targeting strategy for VDR ablation.
